# Preventive effects of royal jelly against anaphylactic response in a murine model of cow’s milk allergy

**DOI:** 10.1080/13880209.2017.1383487

**Published:** 2017-10-05

**Authors:** Malika Guendouz, Abir Haddi, Hadria Grar, Omar Kheroua, Djamel Saidi, Hanane Kaddouri

**Affiliations:** Laboratory of Physiology of the Nutrition and Food Safety, Department of Biology, Faculty of Natural and Life Sciences, University of Oran 1 Ahmed Ben Bella, Oran, Algeria

**Keywords:** β-Lactoglobulin, Balb/c, immunomodulator, Ussing chamber, histamine

## Abstract

**Context:** Royal jelly (RJ) has long been used to promote human health.

**Objective:** The current study investigated the preventive effects of RJ against the development of a systemic and intestinal immune response in mice allergic to cow’s milk proteins.

**Materials and methods:** Balb/c mice treated orally for seven days with RJ at doses of 0.5, 1 and 1.5 g/kg were sensitized intraperitoneally with β-lactoglobulin (β-Lg). Serum IgG and IgE anti-β-Lg were determined by an enzyme-linked immunosorbent assay (ELISA). Plasma histamine levels, symptom scores and body temperature were determined after *in vivo* challenge to β-Lg. Jejunums were used for assessment of local anaphylactic responses by an *ex vivo* study in Ussing chambers and morphologic changes by histological analysis.

**Results:** RJ significantly decreased serum IgG (31.15–43.78%) and IgE (64.28–66.6%) anti-β-Lg and effectively reduced plasma histamine level (66.62–67.36%) (*p* < 0.001) at all the doses tested. Additionally, no clinical symptoms or body temperature drops were observed in RJ-pretreated mice. Interestingly, RJ significantly reduced (*p* < 0.001) intestinal dysfunction by abolishing the secretory response (70.73–72.23%) induced by sensitization and prevented length aberrations of jejunal villi by 44.32–59.01% (*p* < 0.001).

**Discussion and conclusions:** We speculate that using RJ may help prevent systemic and anaphylactic response in allergic mice. These effects may be related to its inhibitory effects on the degranulation of mast cells.

## Introduction

Cows’ milk allergy (CMA) is the most common cause of food allergy in infants and children, affecting approximately 2–3% of the general population (Moneret-Vautrin et al. 2001; Skripak et al. 2007). CMA generally corresponds to an inappropriate immune response characterized by a disruption of the Th1/Th2 balance toward a Th2 profile that results in the production of immunoglobulin E (IgE) specific for cow’s milk proteins. Patients with CMA may be sensitized to various proteins, mainly β-lactoglobulin (β-Lg) and casein (Wal 1998).

Food allergy is treated by avoidance diets in order to prevent anaphylactic reactions and to cure chronic associated symptoms. Nevertheless, eviction significantly alters the quality of life. It does not prevent the development of sensitization with age, and is implicated in increasing the clinical reactivity, resulting in an increased severity or a lower reactogenicity level (Flinterman et al. 2006; Moneret-Vautrin 2011). It is no longer a sufficient security for the most allergic subjects. The majority of severe allergies are IgE-dependent, which justifies the institution of oral or sublingual immunotherapies (OIT, SLIT). However, severe adverse events during this treatment are not uncommon. Whether OIT leads to a sustained, robust development of tolerance in patients has not yet been thoroughly investigated (Trendelenburg and Blümchen 2016).

Furthermore, hypoallergenic infant formulas are commonly used for genetically predisposed children and infants diagnosed with cow’s milk allergy. The most common are the partially hydrolysed infant formulas, prepared from different fractions such as whole milk, whey proteins or caseins, extensively hydrolysed and soy-based formulas (Walker-Smith 2003; Isolauri and Sampson 2004). Although these alternatives are not always adequate and may lead to cross-reactions (Addou et al. 2016) and other dangers that are caused by phytoestrogen content (Agostoni et al. 2006). As there is no effective treatment for food allergy, novel approaches are needed. There is increasing evidence supporting the use of traditional medicine (Li and Brown 2009).

Nowadays, one of the most exciting tracks in traditional medicine is the administration of immunomodulatory substances to treat many illnesses such as Chinese herbs FAHF-2 (Srivastava et al. 2005), German chamomile (Chandrashekhar et al. 2011) and some of the pharmacologically active components present in royal jelly (RJ) (Okamoto et al. 2003; Fujii et al. 2007; Vucevic et al. 2007).

Royal jelly, as the principal food source of the queen honeybee and young larvae, is produced by the hypopharyngeal and mandibular glands of young worker bees, to feed young larvae and the adult queen bee (Kanbur et al. 2009). RJ is composed of proteins (12–15%), sugars (10–16%), lipids (3–6%), vitamins and free amino acids (Nagai and Inoue 2004; Ramadan and Al-Ghamdi 2012). Royal jelly has been demonstrated to have several physiological activities. However, in the literature, reactions induced by RJ are reported in subjects allergic to pollen, honey and propolis (Lombardi et al. 1998; Harada et al. 2011). Even if numerous studies have shown different pharmacological activities of RJ, such as vasodilation and hypotension (Shinoda et al. 1978), hypoglycaemic (Münstedt et al. 2009), hypocholesterolemic (Viuda-Martos et al. 2008; Chiu et al. 2017), antimicrobial (Fontana et al. 2004), anti-inflammatory (Kohno et al. 2004), antioxidant and immunomodulatory effect (Sver et al. 1996; Nagai et al. 2006; Fujii et al. 2007; Liu et al. 2008), there is only limited data on its anti-allergic effects available (Okamoto et al. 2003).

In this report, we investigate the preventive effects of RJ on the development of a systemic and intestinal immune response in a mouse model of cow’s milk allergy.

## Materials and methods

### Chemicals

Royal jelly was obtained from honeybee keepers of the plain of Mitidja located in North Algeria center in April 2011 and stored at −20 °C, β-Lg, the diamine-orthophenylene, anti-mouse IgG biotinylated, extravidin peroxidase, Tween 20, di-potassium hydrogeno-phosphate, magnesium chloride, potassium phosphate, poly(vinyl alcohol) and H_2_O_2_ were purchased from Sigma (St. Louis, MO). Alum [Al(OH)_3_], sulphuric acid, disodium hydrogeno-phosphate, calcium chloride, haematoxylin–eosin (H&E) stains were purchased from Merck (Darmstadt, Germany). Potassium chloride and sodium chloride were purchased from Prolabo (Paris, France); IgE biotinylated monoclonal specific antibody (Pharmingen, France). All materials and instruments of Ussing chamber were purchased from Physiologic Instruments (San Diego, CA).

### Animals and immunization protocol

Female mice Balb/c aged of 3–4 weeks old and weighing (19.50 ± 0.25) g obtained from Pasteur Institute, Algiers, Algeria, were kept under proper conditions of ambient temperature and adequate humidity and divided into five groups (*n* = 20 per group). Four groups received by gavage the RJ for seven days at the respective doses of 0 g/kg (positive control), 0.5, 1 and 1.5 g/kg before immunization with the β-Lg. Group 5 received NaCl (0.9%) with no immunization (negative control). Each group was divided into two subgroups.

Animals were immunized on days 0, 14, 21 and 28 by intraperitoneal injection of β-Lg (10 µg) adsorbed on to 2 mg of alum in 0.1 mL of PBS. Seven days after the final boost, clinical signs were assessed in one sub group of mice which were intraperitoneally challenged with β-Lg (1 mg). The other was used to evaluate systemic antibodies, intestinal anaphylactic response in Ussing chamber and the morphological changes by histological examination. Blood samples were collected from the retro-orbital venous plexus and sera were stored at −20 °C until analysis. Animal’s experiments were approved by the Animal Care and Use Committee of the University of Oran 1 Ahmed Ben Bella.

### *In vivo* hypersensitivity response to β-lactoglobulin

*In vivo* provocation test was performed to evaluate the anaphylactic symptoms in mice 30 min after intraperitoneal challenge with 1 mg of β-Lg/mouse without adjuvant. The symptoms were quantified by using the following previously reported scoring system (Li et al. 1999): 0: no symptoms; 1: scratching and rubbing around the nose and head; 2: puffiness around the eyes, pillar erect, reduced activity and/or decreased activity with decreased activity with increased respiratory rate; 3: wheezing, laboured respiration, cyanosis around the mouth and the tail; 4: no activity after prodding or tremor and convulsion; 5: death.

### Measurement of body temperature

Body temperature was measured 30 min after intraperitoneal β-Lg challenge using a rectal probe.

### Dosage of IgG and IgE anti-β-Lg by ELISA

Specific anti-β-Lg IgG and IgE levels were assayed in serum samples using a colorimetric enzyme-linked immunosorbent assay (ELISA). A multi-well microtiter plate (Maxisorp; Nunc, Roskilde, Denmark) was coated with 100 μL of β-Lg (10 µg/mL) and incubated overnight at 4 °C. After that the plates were washed with phosphate buffered saline containing 0.05% Tween 20, pH 7.4 (ELx50 Auto Strip washer, BioTEK Instruments, Winooski, VT). Residual free binding sites were blocked with 250 μL/well with 1% of PVA/PBS 1/10 at 37 °C for 1 h. After washing, 100 μL of serially diluted sera (1/10^2^ to 1/10^7^) were added in duplicate to each well and incubated at 37 °C for 2 h. Plates were then washed and incubated for 1 h at 37 °C with 100 µL of biotinylated polyclonal-specific antibody for IgG (1/20,000) (Sigma, B9904, Labège, France) or of biotinylated monoclonal specific antibody (Pharmingen, Le Pont-de-Claix, France) for IgE (553387/R1915). Plates were left at 37 °C of temperature for 1 h and 30 min. The plates were again washed and 100 μL of extravidin-peroxidase (E2886, Sigma, Labège, France) (1:5000) were added to the wells. The plates were left for 30 min at 37 °C of temperature. After further washing, peroxidase activity has been assayed by addition of 50 μL H_2_O_2_ (30%, 0.25 mL/L) associated with diamine-orthophenylene (0.5 mg/mL) in 0.05 M citrate buffer, pH 5.1. The stained wells were kept in the dark at room temperature for 30 min. The reaction has been stopped with 2 N H_2_SO_4_ (50 μL/well), then absorbance was measured at 492 nm with an automated ELISA reader (ELx 800 universal microplate reader, BioTEK Instruments, Winooski, VT). Positive titers were given as the last dilution with an optical density above the background.

### Determination of plasma histamine levels

Plasma histamine levels were obtained 30 min after intraperitoneal β-Lg challenge. Blood samples collected in tubes containing EDTA were centrifuged at 1600×*g* for 20 min, then plasma was collected and kept at −20 °C until assay. The plasma histamine was measured by using an Enzyme Immunoassay (EIA) kit (A05890-96 wells; SPI-BIO; BertinPharma, Montigny-le-Bretonneux, France). This EIA kit functions on the principle of competition between unlabelled, derivatized histamine and acetylcholinesterase (AChE) linked to histamine (tracer) for a limited number of specific mouse anti-histamine antibody sites. The absorbance was read at 405 nm on a microplate absorbance spectrophotometer. The concentration of histamine in samples is obtained by comparison with a standard curve as described by the manufacturer.

### *Ex vivo* experiments (Ussing chamber assay)

The effect of specific sensitization to β-Lg on the intestinal response of Balb/c mice treated orally with RJ was analysed *ex vivo*.

### Tissue preparation

Seven days after the last immunization, mice were anesthetized by intraperitoneal injection of sodium penthothal (50 mg kg**^−^**^1^). A segment of jejunum (10–20 cm) was removed and placed in ice-cold oxygenated isotonic Ringer solution containing (in mM): 115 NaCl, 25 NaHCO_3_, 1.2 MgCl_2_, 1.2 CaCl_2_, 2.4 K_2_HPO_4_ and 0.4 KH_2_PO_4_. The pH of this solution was 7.40 at 37 °C when bubbled with the 95% O_2_–5% CO_2_ mixtures used to circulate the chamber fluid.

### Measurement of mucosal ion transport

Jejunal segments were opened longitudinally along the anti-mesenteric border and placed in ice cold oxygenated solution. Each jejunal sheet without any visible Peyer’s patch was mounted in Ussing chamber system with an exposed surface area of 0.2 cm**^2^** and maintained at 37 °C by water-jacketed reservoirs. The tissue sheets were independently bathed on the serosal and mucosal surface with 5 mL of Ringer solution. The tissues were short-circuited by an automatic voltage clamp device (model VCC MC8; Physiologic Instruments, San Diego, CA) and short circuit current (Isc, µA/cm**^2^**) was monitored at interval as an indication of net active ion transport. Tissues were allowed to equilibrate for 20 min period. Then, basal Isc and conductance (*G*, mmho/cm**^2^**) were recorded using Acquire & Analyse 2.3 software (Physiologic Instruments, San Diego, CA). Changes in electrophysiological parameters were determined by measuring changes in Isc and G after addition of 60 μg of β-Lg (or, in some case, ovalbumin (OVA) as non-specific antigen control) to the serosal side. At the end of the experiments, tissue viability was assessed by addition of 10 mM glucose to the mucosal side.

### Histological examination

To investigate the effect of RJ on intestinal changes induced by immunization, a histological study was performed according to Hould (1984). A representative sample of jejunal tissue was taken immediately and was immersed in 10% buffered formalin solution pH 7.2, after which they were dehydrated through graded alcohols to xylene and then embedded in paraffin. Sections were cut at 4 µm and stained with H&E and examined by light microscopy (Optica Axiom 5000, Beijing, China).

### Length of villi

The measurements of the length of villi were taken using a micrometre eyepiece. Length of villi was expressed in μm.

### Statistical analysis

Data are presented as mean ± standard error of mean (SEM). Comparisons between groups were performed by using analysis of variance (ANOVA) or unpaired Student’s *t*-test where appropriate. A *p* value of <0.05 was considered statistically significant.

## Results

### *In vivo* hypersensitivity response to β-Lg

#### Provocation tests: clinical signs

**Figure 1.**
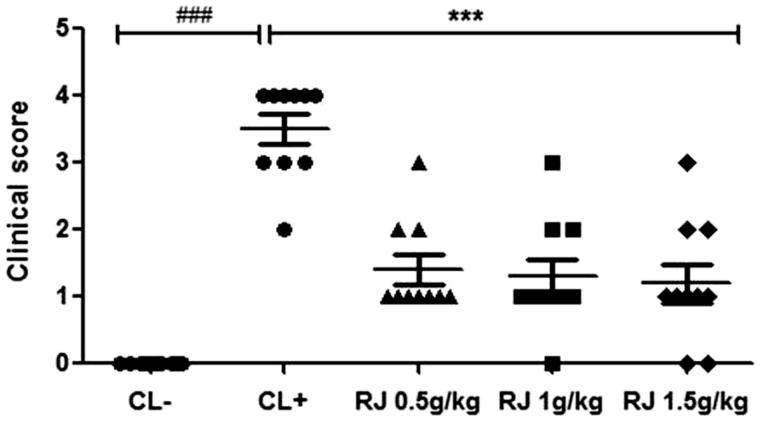
Clinical signs observed in mice following challenge test with the allergen (β-Lg). Data are mean ± SE (standard error). (###*p* < 0.001 compared with unsensitized mice (CL−). ***p < 0.001 compared with positive control mice (CL+); *n* = 10 per group).

Clinical symptoms associated to hypersensitivity reactions were scored 30 min after the intraperitoneal challenge (Figure 1). As shown in Figure 3, sensitized mice (CL+) developed clinical signs immediately after the intraperitoneal challenge with β-Lg. Symptoms were mostly related to respiratory distress and a reduction of general activity. However, control mice (CL−) showed no clinical signs. Similarly, oral administration of RJ for seven days showed few clinical scores or no anaphylactic responses in sensitized mice.

#### Body temperature

**Figure 2.**
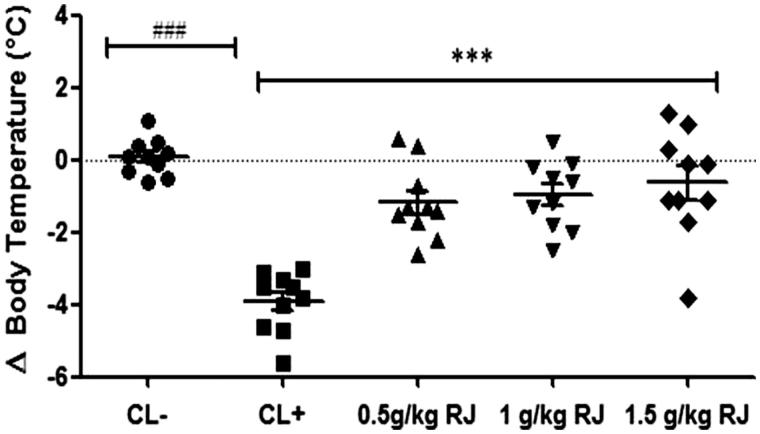
Body temperature of Balb/c mice after β-Lg challenge. Rectal temperatures were measured 30 minutes after IP challenge with β-Lg. Data are mean ± SE (standard error). (###*p* < 0.001 compared with unsensitized mice (CL−). ***p < 0.001 compared with positive control mice (CL+); *n* = 10 per group).

Anaphylactic shock upon allergen challenge is accompanied by a drop in the body temperature. In our study, a rapid decline in body temperature was observed in sensitized mice (positive control) which reached its minimum at 30 min after the intraperitoneal challenge with β-Lg. In contrast, no drop in body temperature was observed in sensitized mice pretreated with RJ at different doses (0.5, 1 and 1.5 g/kg) (Figure 2).

#### Plasma histamine level after intraperitoneal β-Lg challenge

**Figure 3.**
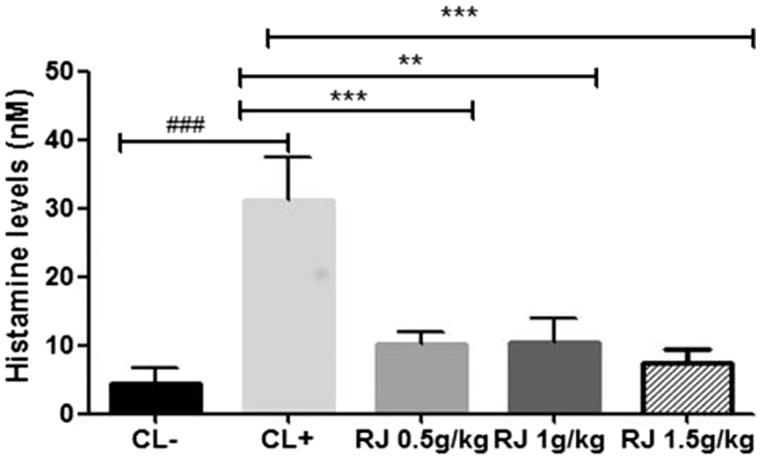
Histamine concentration was determined by enzyme immunoassay kit and represented as plasma concentration (nM). Data are mean ± SE (standard error). (###*p* < 0.001 compared with unsensitized mice (CL−). **p < 0.01; ***p < 0.001 compared with positive control mice (CL+); *n* = 10 per group).

In order to evaluate the intensity of the elicitation phase of the allergic response, plasma histamine levels were measured by an EIA kit after the i.p. challenge with allergen. The results indicated that the oral administration of RJ at different doses for seven days caused a significant decrease in histamine levels (*p* < 0.001) compared to positive control mice (CL+) (Figure 3).

#### Effect of royal jelly administration on IgG and IgE anti-β-Lg levels

To control animal sensitization level, specific antibody responses were assessed using a colorimetric ELISA.

**Figure 4.**
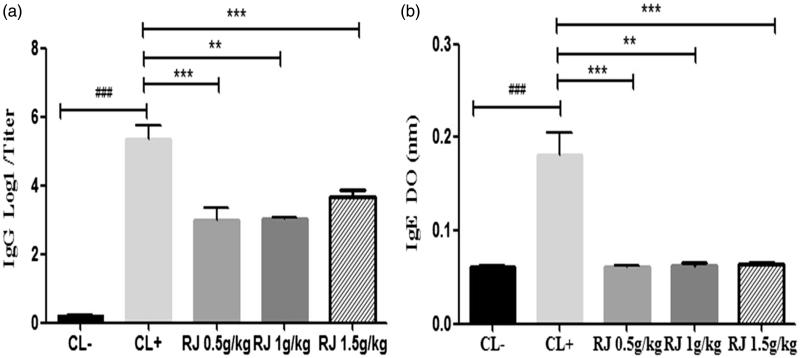
Effect of royal jelly administration on IgG (a) and IgE (b) titers in response to β-Lg sensitization. Data are mean ± SE (standard error). (###*p* < 0.001 compared with unsensitized mice (CL−). **p < 0.01; ***p < 0.001 compared with positive control mice (CL+); *n* = 10 per group).

A clear increase in titers serum IgG and IgE anti-β-Lg was observed in the CL + group (*p* < 0.001) as compared with unsensitized mice (CL−) (Figure 4). Moreover, oral administration of RJ at different doses (0.5, 1 and 1.5 g/kg) for seven days caused a significant decrease in IgG (*p* < 0.001) (Figure 4(a)) and IgE levels (*p* < 0.01) (Figure 4(b)) in sensitized mice when compared to positive control mice (CL+). However, this response was not affected by the dose of RJ used.

#### Measurement of mucosal ion transport

**Figure 5.**
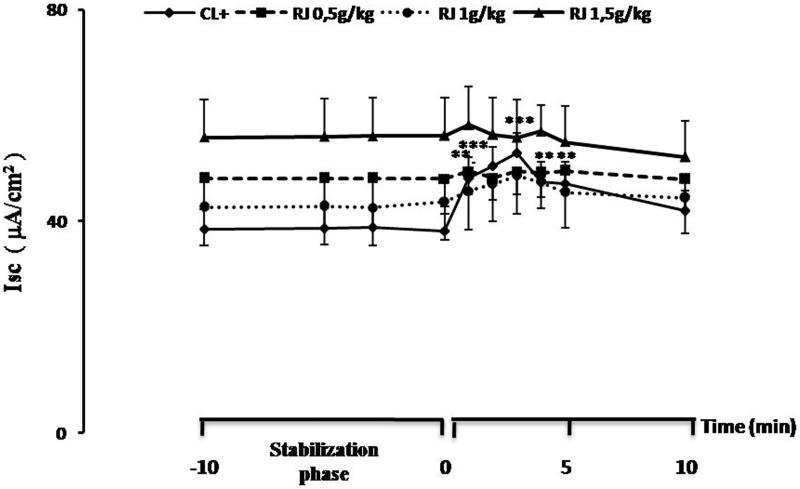
Effect of β-Lg on the short circuit current (Isc) in Ussing chamber measured in mouse jejunal fragments sensitized intraperitoneally with β-Lg previously treated or not with royal jelly. Data are expressed as mean ± SE (*n* = 10) (**p < 0.01 and ***p < 0.001).

**Figure 6.**
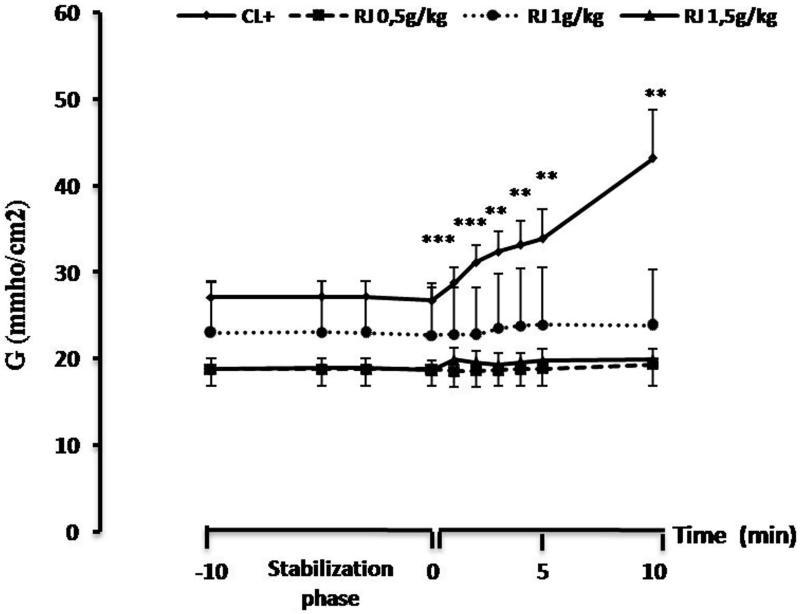
Effect of β-Lg on conductance (*G*) in Ussing chamber measured in mouse jejunal fragments sensitized intraperitoneally with β-Lg previously treated or not with royal jelly. Data are expressed as mean ± SE (*n* = 10) (**p < 0.01 and ***p < 0.001).

Epithelial electrophysiological studies performed using Ussing chamber were employed to assess the effect of challenge to β-Lg on jejunal response of sensitized mice treated orally with RJ. After the electrical parameters were stabilized, β-Lg was added to the serosal side. Compared to negative control mice, an increased Isc was observed in jejunums of sensitized mice. The basal values change from 38.17 ± 3.21 to 54.78 ± 3.55 µA/cm^2^ (ΔIsc = 16.60 ± 1.13, *p* < 0.001) (Figure 5) (Table 1). The increased Isc response was correlated with a gradual time-dependent increased tissue conductance which is a measure of the integrity of tight junctions. The conductance (*G*) values change from 26.79 ± 1.97 to 43.20 ± 5.56 mmho/cm^2^ (Δ*G* = 16.41 ± 3.83, *p* < 0.001) (Figure 6) (Table 1) in sensitized mice (positive control group). However, β-Lg challenge did not produce any significant alteration of electrical parameters in the jejunum of sensitized mice treated orally with RJ regardless of the dose used (Figures 5 and 6) (Table 1).

**Table 1.** Effect of royal jelly on Isc (a) and conductance (b) values after β-Lg challenge.

	Isc (µA cm^−2^)		
	*T*_0_	*T*_MAX_	ΔIsc
(a)			
Negative control	34.80 ± 3.61	37.16 ± 3.68	2.35 ± 0.56
Positive control	38.17 ± 3.21	54.78 ± 3.55	16.60 ± 1.13***
RJ 0. 5 g/kg	48.03 ± 4.73	53.00 ± 4.73	4.97 ± 1.05###
RJ 1 g/kg	43.75 ± 6.59	48. 59 ± 6.09	4.86 ± 2.19###
RJ 1.5 g/kg	56.16 ± 7.23	60.78 ± 5.45	4.61 ± 2.47###
	*G* (mmho/cm^2^)		
	*T*_0_	*T*_MAX_	Δ*G*
(b)			
Negative control	21.54 ± 1.98	22.73 ± 1.95	1.18 ± 0.21
Positive control	26.79 ± 1.97	43.20 ± 5.56	16.41 ± 3.83***
RJ 0.5 g/kg	18.83 ± 1.94	20.50 ± 2.18	1.67 ± 0.71###
RJ 1 g/kg	22.83 ± 5. 67	24.33 ± 6.28	1.50 ± 0.67###
RJ 1.5 g/kg	18.70 ± 1.24	20.98 ± 1.26	2.27 ± 0.68###

The increase in Isc (ΔIsc) and *G* (Δ*G*) is the difference between the peak value after β-Lg challenge and the baseline value.

Values represent mean ± SE (standard error); *n* = 10 mice per group.

*** *p* < 0.001 compared with negative control (CL−).

### *p* < 0.001 compared with positive control (CL−).

#### Histological study

**Figure 7.**
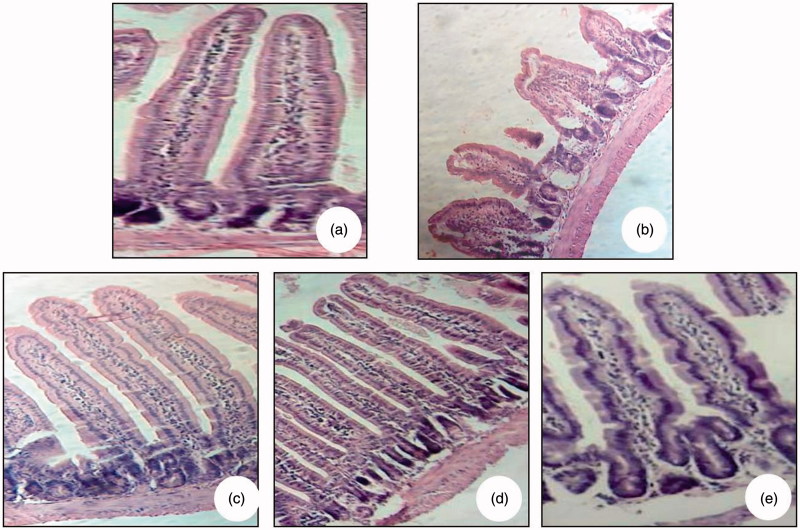
Light microscopy (*G* × 10) showing intestinal villi stained with haematoxylin–eosin. Jejunal tissues were obtained from naive mice (CL−) (a), β-Lg-sensitized mice (CL+) (b) and mice received royal jelly for seven days at the respective doses of 0.5 (c), 1 (d) and 1.5 g/kg (e) and then sensitized intraperitoneally with β-Lg.

To investigate the effect of oral administration of RJ on the microscopic changes of intestine in sensitized mice, jejunal segments of experimental and control animals were subjected to histological examination. The intestinal mucosa of sensitized mice presented a massive increase in the inflammatory infiltrate in the lamina propria, a partial villous atrophy and a significant crypt hyperplasia (Figure 7(b)). However, no effect on jejunal structure was shown in mice pretreated with RJ and sensitized intraperitoneally with β-Lg (Figure 7(c–e)).

#### Effect on villus length

**Figure 8.**
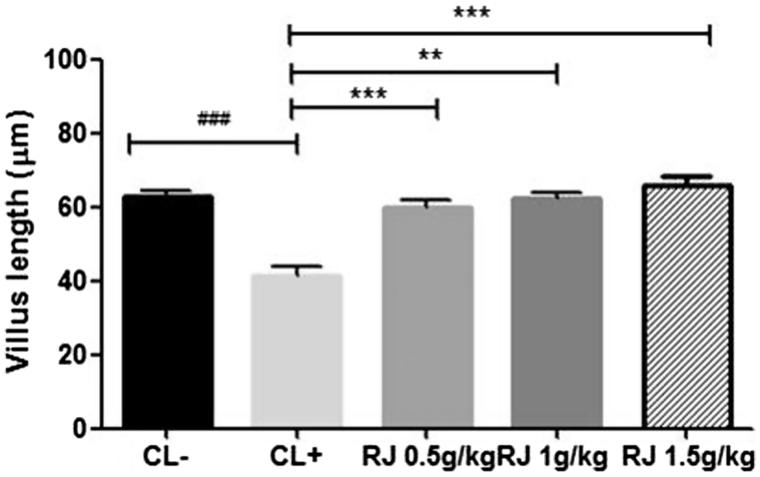
Evaluation of the villus height fragments of jejunum of mice received royal jelly for seven days at the respective doses of 0 (positive control), 0.5, 1 and 1.5 g/kg and then sensitized intraperitoneally with β-Lg. Data are mean ± SE (standard error). (###*p* < 0.001 compared with unsensitized mice (CL−). **p < 0.01; ***p < 0.001 compared with positive control mice (CL+); *n* = 10 per group).

The mean values of villus length determined from sections of jejunum in the negative and positive control groups are respectively (62.75 ± 1.72 μm) and (41.50 ± 2.36 µm) with *p* < 0.001. In experimental groups, length of villi measured is similar to that of the negative control group. The effects of the administration of RJ at different doses (0.5, 1 and 1.5 g/kg) for seven days and intraperitoneal β-Lg sensitization are shown in Figure 8.

## Discussion

In this study, Balb/c mice were used as a model of cow’s milk allergy, as described by Adel-Patient et al. (2005). After intraperitoneal immunization with β-Lg, the sensitization level was assessed with an immuno-enzymatic method. We have shown that β-Lg immunization leads to a significant production of IgG and IgE anti β-Lg. Moreover, as shown by *in vivo* test challenge, β-Lg induced acute clinical symptoms. These results agree well with those obtained by Adel-Patient et al. (2005), who demonstrated that intraperitoneal immunization using alum as an adjuvant induce the production of specific IgE and IgG1, which results from allergens specific Th2 cell response, as demonstrated by the IL4 and IL5 secretions by splenocytes.

The production of specific IgE and IgG is significantly inhibited by the oral administration of RJ and no clinical symptoms were observed. Clinical signs can appear following a type 1 hypersensitivity response after a release of inflammatory mediators. Among these mediators, histamine acts as potent activator of the inflammatory response in epithelial cells. Indeed, histamine release can be a criterion for the diagnosis of food allergy (Crockard and Ennis 2001). Increase in histamine levels reflects mast cell degranulation and increases vascular permeability, resulting in aggravation of allergic reactions. Many reports suggest that allergic reactions, therefore, can be reduced by the suppression of histamine release. In light of this, our results showed that RJ intake significantly decreased the plasma levels of histamine.

Several possibilities have been raised as the underlying mechanisms of the immunomodulatory and anti-allergic effect of RJ, Vucevic et al. (2007) suggested a specific role for RJ in regulating T-cell proliferation, histamine production, Th2 cytokine production and the antibody IgE and IgG production. Okamoto et al. (2003) showed that intraperitoneal administration of major RJ protein 3 (MRJP3) into OVA/alum-immunized mice inhibited serum anti-OVA allergen specific IgE and IgG1 levels. It was also reported that RJ suppressed antigen-specific IgE production and histamine release from mast cells in association with the restoration of macrophage function and improvement of Th1/Th2 cell responses in immunized mice (Oka et al. 2001).

The typical responses of the intestinal epithelium to allergens are an increase in watery diarrhoea due to the stimulation of chloride secretion, and increased protein inward permeability from lumen to blood (Baron et al. 1988). The Isc, transepithelial voltage potential (Vt) and conductance (*G*) measurements in Ussing chamber studies have shown that electrogenic Cl^−^ secretion is not only important to normal digestive physiology, but is also a marker of enterotoxic and inflammation-mediated secretory diarrhoea (Clarke 2009).

In this study, compared with naïve mice (negative control), Ussing chambered-intestinal segments from sensitized mice (positive control) responded to serosal β-Lg challenge with an increase in Isc values. This increase is probably due to a secretory response and might well reflect local anaphylactic responses. β-Lg challenge elicited a very rapid Isc response that began approximately 1 min after the antigen addition and maximally expressed within 2–3 min. These results confirm our previous studies on jejunal responses to bovine β-Lg in infants during the active phase of cow milk allergy (Saidi et al. 1995) and in animal models (Negaoui et al. 2009) showing that challenge with β-Lg of jejunal fragments induced a significant increase in Isc due to electrogenic chloride secretion.

The increased Isc response was correlated with a gradual time-dependent increased tissue conductance which is a measure of the integrity of tight junctions. This suggested an impairment of intercellular junctions of intestinal epithelium and implies that a local anaphylactic reaction occurs when β**-**Lg interacts as an allergen with the intestinal immune system of β-Lg-sensitized mice. To verify the β-Lg-specific response, the tissues were challenged with OVA and no changes in the Isc were observed (data not shown).

Several studies have reached similar conclusions regarding the effect of β-Lg on the epithelium conductance, concluding that immunization alters the tight junction and increases the para-cellular permeability of the intestinal epithelium (Terpend et al. 1999; Negaoui et al. 2009; El Mecherfi et al. 2015; Grar et al. 2015).

In contrast, no significant changes in the values of the Isc were observed after β-Lg challenge of mice jejunums that have received RJ at 0.5, 1 and 1.5 g/kg then immunized with β-Lg. As shown by Isc values which remain stable and close to the control group, our results suggest the absence of immediate local anaphylactic reaction when milk proteins interact with the elements of the immune system of the intestinal epithelium.

Mast cells are important effectors cells of type I allergic reactions as well as of other inflammatory process (Hagenlocher and Lorentz 2015). The role of mast cells in the regulation of epithelial ion secretion has been well studied, and it has been clearly established that their mediators such as histamine and prostaglandins can act via specific receptors on the intestinal epithelium to initiate chloride ion secretion (Crowe and Perdue 1992). These chemical mediators cause the characteristic symptoms of allergy (Kawa 2012).

The mechanism by which RJ inhibited anaphylaxis induced by β-Lg sensitization could not be explained from the present study but it is possible that RJ, as showed by Oka et al. (2001) in DNP-KLH mice, suppressed histamine release from mast cells by acting synergistically with immunocytes, e.g., restoration of macrophage function and improvement of Th cell responses, resulting in the suppression of vascular permeability.

Morphological changes in the epithelium due to the immunologic reaction were underscored by histological results demonstrating villi atrophy and greater increase of mucosal inflammatory cells in jejunum. Several published studies showed that food allergy in mice is characterized by villus atrophy and goblet cell hyperplasia, as well as infiltration of IgE-positive mast cells performing degranulation in the jejunum and increased-histamine release (Nakajima-Adachi et al. 2006; Sun et al. 2008; Huang et al. 2010; Chen et al. 2011; Grar et al. 2015). Usually, IgE-dependent food allergy reactions affect one or more target organs such as skin, respiratory tract, gastrointestinal tract and cardiovascular system (Sicherer 2002).

Treatment with RJ was found to diminish the harmful effects of β-Lg immunization. RJ has been shown to play a central protective role against tissue damage (Ghanbari et al. 2016a, 2016b). Although, it has been considered that RJ might exert the benefits through its antioxidant features up to now, the exact mechanism of its effect is yet unknown. Indeed, RJ contains many polyphenolic compounds collected by bees from the plants where they gather nectar (Fiorani et al. 2006). These polyphenolic compounds in their many forms are the main components responsible for the functional properties associated with many foods, such as antioxidant capacity (Kerem et al. 2006; Almaraz-Abarca et al. 2007) and anti-inflammatory capacity (Wu et al. 2006).

This investigation is in accordance with the study conducted by Kaynar et al. (2012), who showed that oral administration of RJ has a decreasing effect on the methotrexate (MTX)-induced intestinal damage and it has a suppressive effect on MTX-induced oxidative stress by means of increasing antioxidant enzyme activity. Recently, many authors have reported that the antioxidant effect is important for wound healing (Lee et al. 2012; Deniz et al. 2013).

## Conclusions

Oral administration of RJ inhibited serum anti-β-Lg IgE and IgG as well as histamine plasma levels in immunized mice and clearly reduced the intestinal anaphylactic response and histological lesions caused by β-Lg sensitization. These results suggest that RJ may have a beneficial effect on the allergy to cow's milk protein by reducing its symptoms.
